# Neurotensin promotes the progression of malignant glioma through NTSR1 and impacts the prognosis of glioma patients

**DOI:** 10.1186/s12943-015-0290-8

**Published:** 2015-02-03

**Authors:** Qing Ouyang, Xueyang Gong, Hualiang Xiao, Ji Zhou, Minhui Xu, Yun Dai, Lunshan Xu, Hua Feng, Hongjuan Cui, Liang Yi

**Affiliations:** Department of Neurosurgery, Daping Hospital, Third Military Medical University, Chongqing, China; State Key Laboratory of Silkworm Genome Biology, Institute of Sericulture and Systems Biology, Southwest University, Chongqing, China; Department of Neurosurgery, Southwest Hospital, Third Military Medical University, Chongqing, China; Department of Pathology, Daping Hospital, Third Military Medical University, Chongqing, China; Division of Hematology/Oncology, Department of Medicine, Virginia Commonwealth University, Richmond, VA USA

**Keywords:** Glioma, Neurotensin, Proliferation, Invasion

## Abstract

**Background:**

The poor prognosis and minimally successful treatments of malignant glioma indicate a challenge to identify new therapeutic targets which impact glioma progression. Neurotensin (NTS) and its high affinity receptor (NTSR1) overexpression induces neoplastic growth and predicts the poor prognosis in various malignancies. Whether NTS can promote the glioma progression and its prognostic significance for glioma patients remains unclear.

**Methods:**

NTS precursor (ProNTS), NTS and NTSR1 expression levels in glioma were detected by immunobloting Elisa and immunohistochemistry assay. The prognostic analysis was conducted from internet by R2 microarray platform. Glioma cell proliferation was evaluated by CCK8 and BrdU incorporation assay. Wound healing model and Matrigel transwell assay were utilized to test cellular migration and invasion. The orthotopic glioma implantations were established to analyze the role of NTS and NTSR1 in glioma progression *in vivo*.

**Results:**

Positive correlations were shown between the expression levels of NTS and NTSR1 with the pathological grade of gliomas. The high expression levels of NTS and NTSR1 indicate a worse prognosis in glioma patients. The proliferation and invasiveness of glioma cells could be enhanced by NTS stimulation and impaired by the inhibition of NTSR1. NTS stimulated Erk1/2 phosphorylation in glioma cells, which could be reversed by SR48692 or NTSR1-siRNA. *In vivo* experiments showed that SR48692 significantly prolonged the survival length of glioma-bearing mice and inhibited glioma cell invasiveness.

**Conclusion:**

NTS promotes the proliferation and invasion of glioma via the activation of NTSR1. High expression levels of NTS and NTSR1 predict a poor prognosis in glioma patients.

**Electronic supplementary material:**

The online version of this article (doi:10.1186/s12943-015-0290-8) contains supplementary material, which is available to authorized users.

## Background

For the last decade, the improvement of neurosurgery, radiotherapy and chemotherapy have prolonged the survival time of malignant glioma patients. However, the high recurrence rate still results in the high death rate of patients [1]. Vigorous proliferation and extensive invasion make it extremely difficult to completely clear out glioma. The high cellular invasiveness and the residual glioma cells become the sources of recurrence [2]. Inhibition of cellular proliferation and invasiveness has always been a basic strategy to combat malignance. However, the effective therapies that suppress the growth and invasiveness of gliomas are limited, and the underlying mechanisms need to be investigated further.

Neurotenin (NTS) is present in the central nervous system (CNS) and in periphery. High expression level of NTS can be detected in hypothalamus, median eminence, pituitary stalk, substantia nigra, locus coeruleus, raphe nuclei and brainstem structure, especially in amygdale, arcuate nucleus and limbic system which are closely related to psychological activity. However, NTS has the low expression level in cerebral cortex,hippocampus, basal ganglion and thalamus [3,4]. It acts as a neurotransmitter function to inhibit dopaminergic pathways and induce a serial of neurological effects. In the periphery, NTS is mainly secreted by endocrine N-cells of the gastrointestinal tract and plays the role of a neurocrine hormone to regulate the postprandial digestive process. It inhibits gut motility and gastric acid secretions, stimulates the pancreatic and biliary secretions and improves the fatty acid ingestion [5,6].

It has reported that NTS and its high-affinity neurotensin receptor 1 (NTSR1) overexpress in several types of cancer and malignant cell lines. Accumulating evidences also confirm that the activation of NTS/NTSR1 complex results in cancer progression and poor prognosis in breast cancer, malignant pleural mesothelioma, and head and neck squamous cell carcinomas [6,7]. NTSR1 activates at least three major pathways in cancer, which are small GTPases activation inducing cellular mobility, intracellular Ca^2+^ mobilization involving in gene regulation and proto-oncogene serine/threonine-protein kinase/mitogen-activated protein kinase/extracellular signal-regulated kinase (Raf-1/Mek/Erk) cascade inducing cell proliferation [6]. Our previous research found that NTS is highly upregulated in glioma stem cells and promotes the motility of microglia [8]. However, as a neuropeptide in the CNS, the potential biological functions of NTS/NTSR1 and their downstream signaling pathway in glioma are unclear.

Here, we detected the expression levels of NTS and NTSR1 in glioma specimens and investigated the relationship between the expression levels and the patients’ prognosis. The role of the NTS/NTSR1/Erk1/2 signal axis in the proliferation and invasiveness of malignant glioma cells was tested *in vitro*. We established intracranial orthotopic transplantation gliomas in mice, and analyzed the impact of the NTSR1 specific inhibitor SR48692 on the biological behaviors of the glioma cells and the survival time of the glioma-bearing mice. Our results highlighted that NTS promotes the proliferation and invasiveness of malignant glioma cells through NTSR1 and its downstream signaling molecules, leading to Erk1/2 phosphorylation. We firstly reported that high levels of NTS or NTSR1 expression were correlated with a poor prognosis in the glioma patient, which would be a potential target for glioma treatment and need to be further investigated.

## Results

### NTS and NTSR1 expression patterns in human glioma specimens

**Figure 1:**
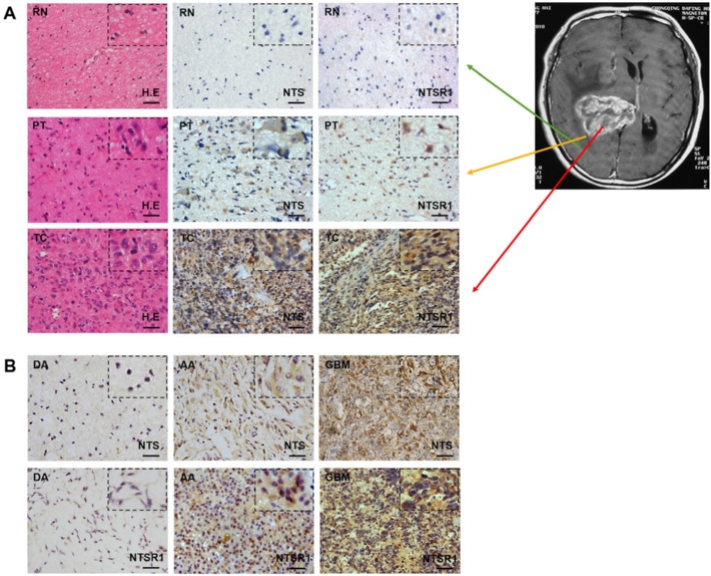
**NTS and NTSR1 expression pattern in gliomas. A**, NTS and NTSR1 expression in tumor core (TC), peritumoral tissue (PT) and relatively normal tissue (RN) around glioma were detected by IHC. Hematoxylin counterstain, scale bar = 50 μm. The images with a larger magnification in the corner of every figure. **B**, NTS and NTSR1 expression in DA, AA and GBM were detected by IHC. Hematoxylin counterstain, scale bar = 50 μm. The images with a larger magnification in the corner of every figure.

We obtained 30 glioma samples of different pathological grades from the clinical glioma sample library in the Neurosurgery Department at Daping Hospital, Third Military Medical University (Additional file 1: Table S1), The peritumoral tissue and the relative normal brain tissue around GBM also collected in fistulization procedure of tumorectomy. The NTS/NTSR1 were detected by immunohistochemistry (IHC) and the results confirmed that NTS/NTSR1 expression were obviously elevated in human glioma compared with the peritumoral tissue and the relative normal brain tissue (Figure 1A).

**Figure 2:**
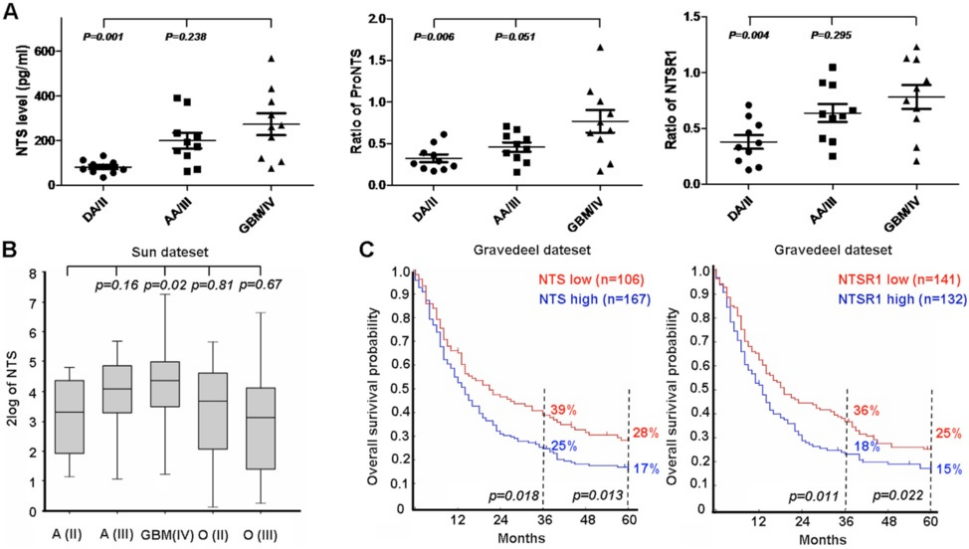
**High NTS and NTSR1 expression indicated poor prognosis in glioma patients. A**, Scatter grams of NTS expression levels, as well as ProNTS and NTSR1 expression quantification of western blot in 30 glioma specimens with different histological grades; the *p* values are indicated. **B**, Box plot of NTS expression levels in different pathological types of glioma and different grades in the Sun dataset; the *p* values compared to the astrocytoma group are indicated. **C**, Kaplan–Meier analysis of overall survival according to NTS and NTSR1 expression for the Gravedeel dataset; *p* values were determined using the log-rank test and were indicated.

Meanwhile, we tested the expression level of NTS and NTSR1 in the 30 glioma samples by IHC and western blot analysis. NTS and NTSR1 showed high levels of expression, particularly in the glioblastoma (GBM) samples. NTS and NTSR1 were mainly located at cytoplasm and cellular membrane of glioma cells (Figure 1B). The IHC quantification showed that the expression level of NTS and NTSR1 was significantly high as the glioma pathological grade was increased (Additional file 1: Table S1 and Additional file 2: Table S2). The western blot analysis of the 30 glioma clinical specimens showed that the expression level of NTS precursor (ProNTS) in GBM was higher than diffuse astrocytoma (DA) (*p* = *0.006*) and anaplastic astrocytoma (AA) (*p* = *0.051*). Consistent with the ProNTS expression in glioma, GBM produced significantly higher NTS level than DA (*p* = *0.001*). Compared with DA, the NTSR1 expression level in GBM increased significantly (*p* = *0.004*) (Figure 2A and Additional file 3: Figure S1).

### High NTS and NTSR1 expression indicated a poor prognosis for glioma patients

The Sun dataset [9] from R2 microarray platform includes 153 glioma cases with different histological grades (grade II, III, and IV). The microarray data have been submitted to the Gene Expression Omnibus (GEO) public database at NCBI (GSE4290). The result of Affymetrix HU133 Plus 2.0 confirmed that increased NTS expression significantly was correlated with advanced tumor stages in the Sun dataset (Figure 2B).

Since NTS and NTSR1 expression was associated with the pathological grade of glioma, we investigated the possibility of NTS or NTSR1 as a prognostic marker for glioma patients. We found that high NTS and NTSR1 mRNA expression indicated a poor outcome in the Gravedeel dataset [10], which includes a cohort of 273 glioma patients (Figure 2C). Kaplan–Meier analysis of the overall survival for this dataset showed that the 3-year survival rates for patients with high expression (167 cases) and low expression (106 cases) of NTS mRNA was 25% and 39% (*p* = 0.018), respectively, and the 5-year survival rates for these patients was 17% and 28% (*p* = 0.013), respectively. Meanwhile, the 3-year survival rates for patients with high expression (132 cases) and low expression (141 cases) of NTSR1 mRNA was 18% and 36% (*p* = 0.011), respectively, and the 5-year survival rates for these patients was 15% and 25% (*p* = 0.022), respectively. We confirmed that high NTS and NTSR1 expression were both associated with poor prognosis, whereas low NTS and NTSR1 expression were associated with good outcome (Figure 2C). The prognostic value of NTS was also verified in Rembrandt database, especially in “Astrocytoma” sub-database. However, NTS had no relationship with the overall survival probabilities of *de novo* GBM patients in TCGA database, but had a significantly negative relationship with their progression-free survival probability (Additional file 4: Figure S2).

### NTS promoted malignant glioma cell proliferation and invasion through NTSR1

**Figure 3:**
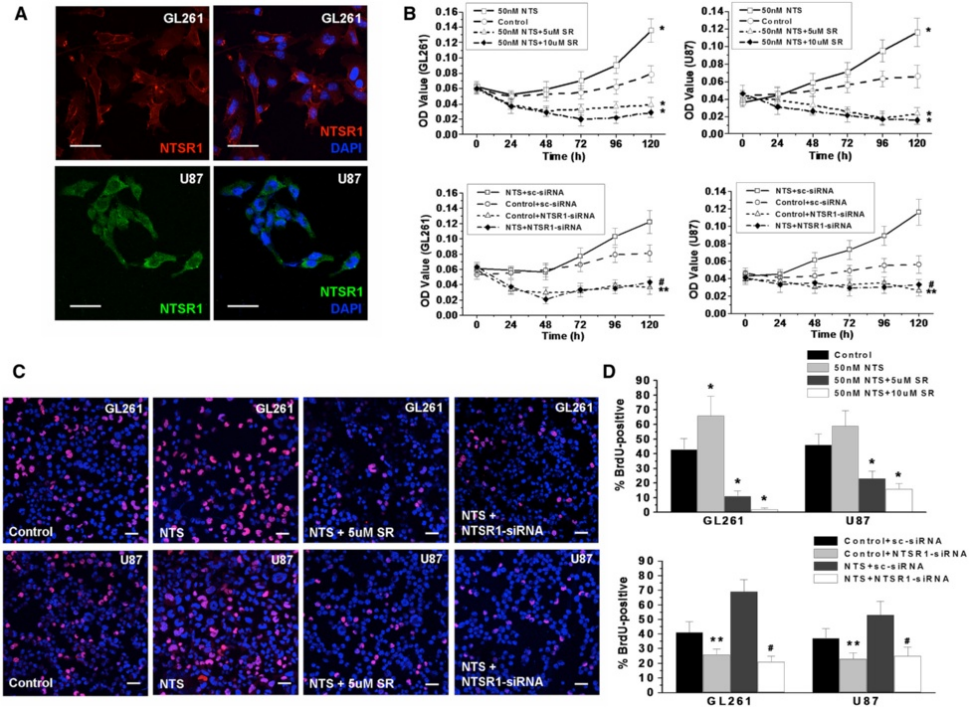
**NTS**/**NTSR1 promoted the proliferation of glioma cells. A**, The expression of NTSR1 in GL261 and U87 glioma cells were detected by immunofluorescence staining. **B**, CCK8 were used to test the effect of NTS, SR48692 and NTSR1-siRNA on the proliferation of GL261 and U87 glioma cells. **C**, Immunofluorescence staining showed BrdU-positive GL261 and U87 glioma cells in the different groups after BrdU incorporation. **D**, The histogram of BrdU incorporation experiments. *p < 0.01 vs. the control group using a two-tailed t test, **p < 0.01 vs. the control + sc-siRNA group using a two-tailed t test, ^#^ *p* < 0.01 vs. the NTS + sc-siRNA group using a two-tailed t test. **A**, **B** Merged images; the nuclei were counterstained with DAPI and are shown in blue. Laser confocal scanning microscopy, scale bar = 20 μm.

To visualize the expression of NTSR1 in glioma cells, we performed immunofluorescence staining in the malignant glioma cell lines GL261 and U87. NTSR1 was very distinctly expressed in GL261 and U87 using both Cy3 and FITC conjugated secondary antibodies and was consistently localized to the membrane (Figure 3A). Both the cell counting kit-8 (CCK8) chromogenic experiment and the bromodeoxyuridine (BrdU) incorporation experiment showed that NTS could promote cell proliferation in serum free medium, whereas SR48692, a specific inhibitor of NTSR1, could significantly inhibit the growth of glioma cells and decrease the number of BrdU-positive cells. The tumor cell growth rates and the percentages of BrdU-positive cells were obviously reduced when cells were treated with increasing concentrations of SR48692 (Figure 3B-D). To further examine the role of NTSR1 in glioma cell proliferation promoted by NTS, we transfected an NTSR1 specific small interfering RNA (siRNA) into the glioma cells. Western blot analysis showed that the expression of NTSR1 in glioma cells was significantly reduced following siRNA treatment (Additional file 5: Figure S3D). Data from the CCK8 chromogenic experiment and the BrdU incorporation experiment demonstrated that the proliferation ability of the NTSR1 depleted cells was significantly inhibited (Figure 3B-D). On the dose of 10 μM of SR 48692, no apoptosis peak could be detected in U87 and GL261 glioma cell lines (Additional file 5: Figure S3C).

**Figure 4:**
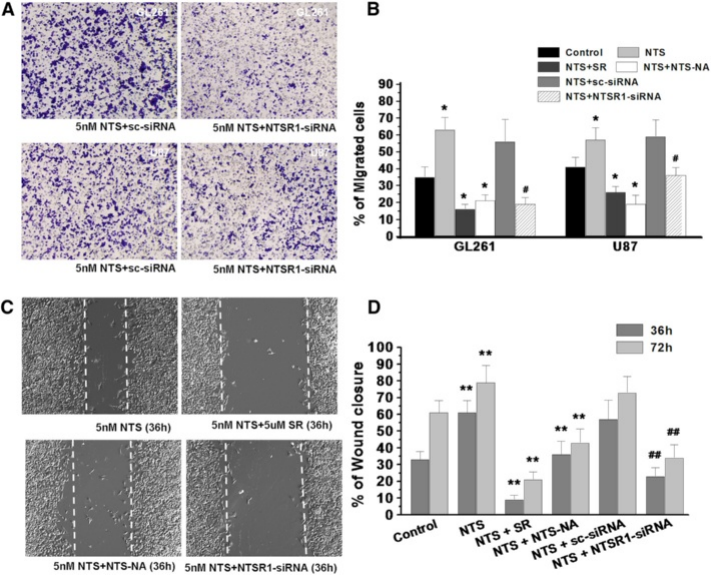
**NTS**/**NTSR1 boosted the migration capacity and invasiveness of glioma cells. A**, Transwell invasion assay showed that the ability of GL261 and U87 glioma cells to invade across the matrigel and membrane. **B**, The proportion of invasive GL261 and U87 glioma cells in all the experimental groups in the transwell experiments. *p < 0.01 vs. the control group using a two-tailed t test, & *p* < 0.01 vs. the NTS group using a two-tailed t test. ^#^ *p* < 0.01 vs. the NTS + sc-siRNA group using a two-tailed t test. **C**, Illustrations of the scratch wounds inflicted by a pipette tip. After 36 hours, the scratch wounds were recolonized by GL261 cells cultured in low-serum medium (0.1% FCS). **D**, The percentage of wound closure by the cells was quantified in the different groups at 36 hours and 72 hours. && *p* < 0.01 vs. the NTS group using a two-tailed t test. **p < 0.01 vs. the control group using a two-tailed t test, ^##^ *p* < 0.01 vs. the NTS + sc-siRNA group using a two-tailed t test.

NTSR1-siRNA treatment inhibited the transwell invasion of GL261 cells and U87 cells when NTS was added (Figure 4A). Approximately 19% ± 3.9% of GL261 cells and 36% ± 4.6% of U87 cells treated with NTSR1-siRNA moved toward the pores of the transwell filters, compared to 56% ± 13.2% of GL261 cells and 59% ± 9.9% of U87 cells when only NTS was added. We also confirmed that SR48692 and NA-NTS treatment significantly impaired transwell invasion of glioma cells compared to the glioma cells of the control group (Figure 4B). In the wound healing assay, the gap size after 36 hours was wider in the cells treated with SR48692 or NA-NTS compared to the control cells (Figure 4C). The gap was reduced by 9% ± 2.7% and 36% ± 7.9% in the treated cells compared to the 61% ± 7.3% gap closure in the cells treated with NTS alone (Figure 4D). Additionally, we confirmed that the wound healing effect of glioma cells transfected with the NTSR1-siRNA was significantly decreased compared to the glioma cells of the control group. In summary, the results from the wound healing and transwell invasion assays confirmed that NTS could promote the migration capacity and invasiveness of GL261 cells and U87 cells.

### NTSR1 activated the phosphorylation of Erk1/2 to promote the proliferation and invasiveness of glioma cells

**Figure 5:**
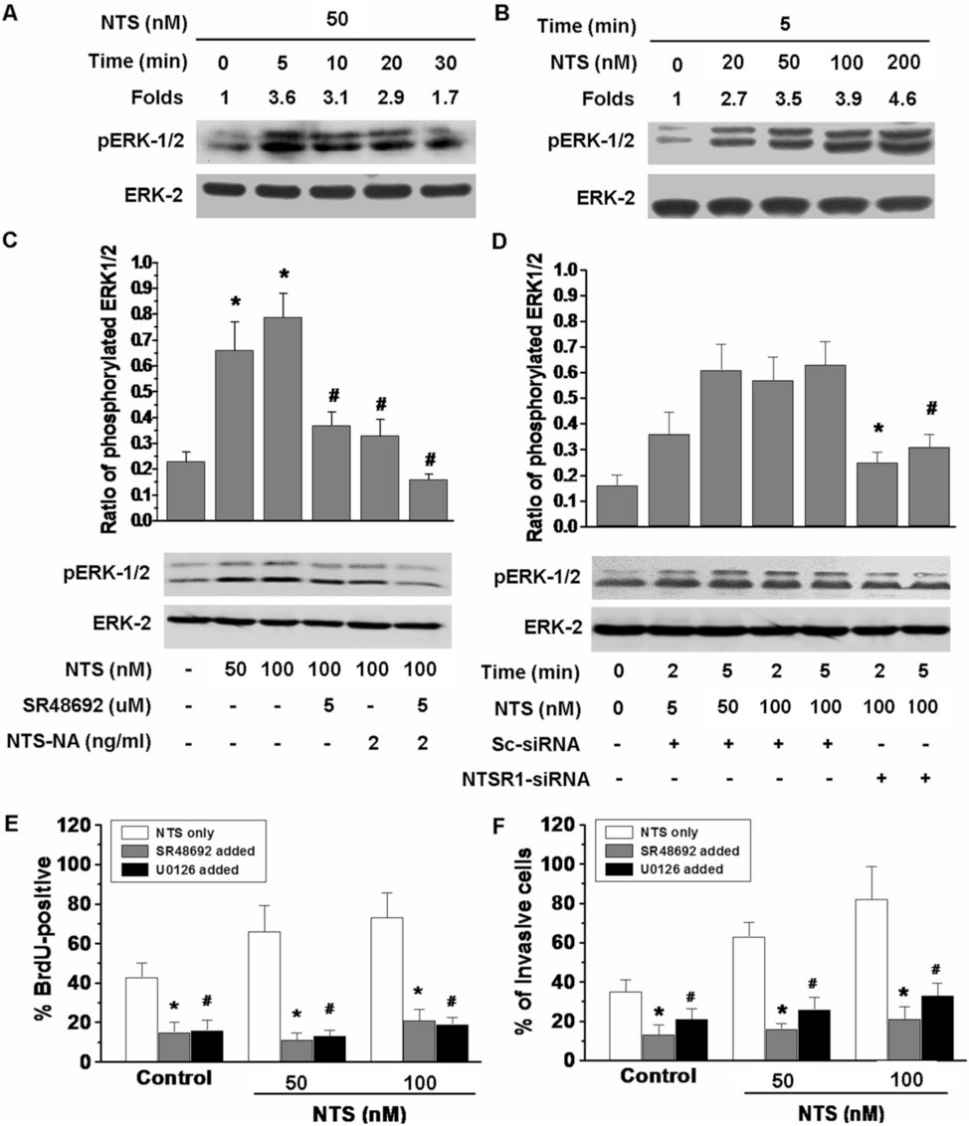
**NTS induced the activation of Erk1**/**2 through NTSR1 in GL261 glioma cells. A**, GL261 glioma cells were stimulated with 50 nM NTS for the indicated times. **B**, Glioma cells were stimulated with NTS at indicated concentrations for 5 min. **C**, GL261 glioma cells were stimulated with NTS for 5 min in the absence or presence of 5 μM SR48692 or 2 ng/ml NTS-NA. *p < 0.01 vs. the control group, ^#^ *p* < 0.01 vs. the 100 nM NTS-stimulated group. **D**, GL261 glioma cells were stimulated with NTS for the indicated times in the presence of Sc-siRNA or NTSR1-siRNA. *p < 0.01 vs. the 100 nM NTS-stimulated group in the presence of Sc-siRNA for 2 min, ^#^ *p* < 0.01 vs. the 100 nM NTS-stimulated group in the presence of Sc-siRNA for 5 min. The cell lysates were evaluated by western blot analysis using antibodies against phospho-Erk1/2. The results were standardized to the total levels of Erk2 and are expressed as the mean ± SEM from at least three independent experiments. **E** and **F**, The percentages of BrdU-positive cells and invasived cells in the presence of SR48692 and the MEK1/2-selective inhibitor U0126. *p < 0.01 vs. the control group, ^#^ *p* < 0.01 vs. the control group.

We found that treatment with 50 nM NTS rapidly and transiently increased the phosphorylation of Erk1/2, with a maximal stimulation after 5 min (Figure 5A). The phosphorylation of Erk1/2 increased along with the increasing concentrations of NTS (Figure 5B). To check whether there were another the NTS-induced proliferation and invasion, we detected molecules and pathways that might mediate the effects of NTS.

We found that Raf-1/Mek/Erk1/2 pathway was activated after NTS stimulation, but not other molecules and pathways, including p15, p16, p38, pAKT, mTOR, pSmad2/3, pJAK2 and pSTAT3 (Additional file 6: Figure S4). The phosphorylation of Erk1/2 could be inhibited by the NTS neutralizing antibody, the NTSR1-selective antagonist SR48692 and NTSR1-siRNA; these results suggested that the elevated phosphorylation of Erk1/2 stimulated by NTS was induced in an NTSR1-dependent manner (Figure 5C, D). Additionally, the MEK1/2-selective inhibitor U0126 reduced the amount of BrdU-positive cells and invasive cells induced by NTS (Figure 5E, F). These results indicated that the above effects of NTS and NTSR1 in glioma cells were induced by the activation of the MEK/ERK signaling pathway.

### NTSR1-selective antagonist SR48692 inhibited glioma progression *in vivo*

**Figure 6:**
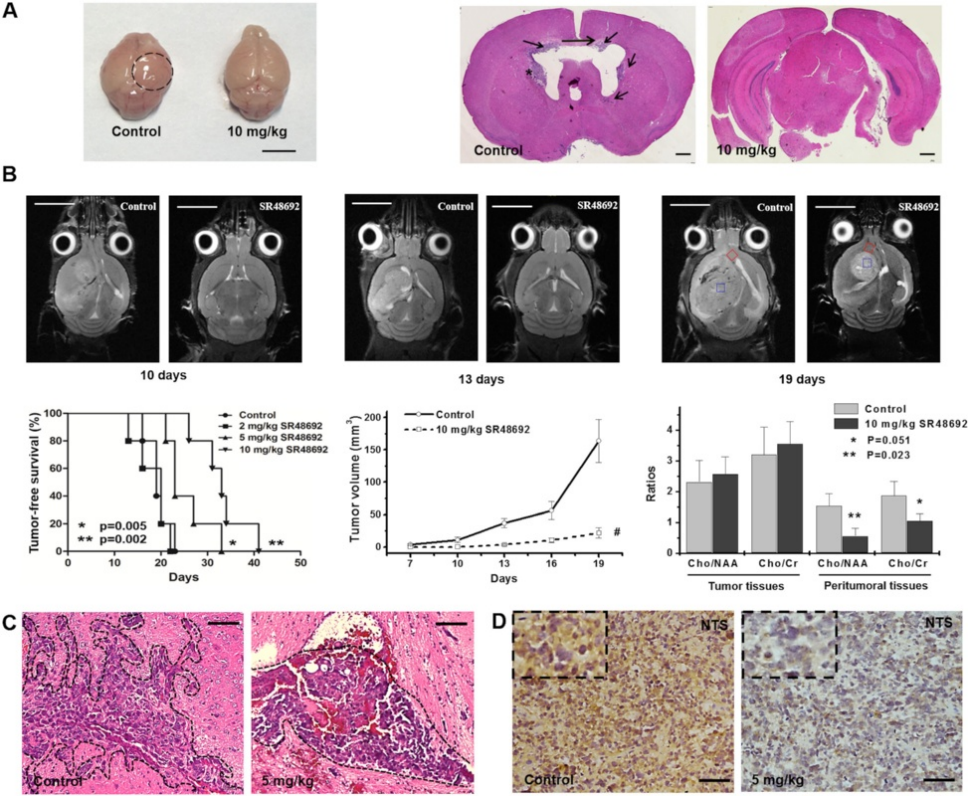
**NTSR1**-**selective antagonist SR48692 inhibited glioma progression** ***in vivo*** **. A**, General observation showed that brain swelling was present in the mice of the control group (dotted black circle) after 10 days of intracranial incubation, scale bar = 1 cm. Glioma progressed and invaded the contralateral tissue during the early stages of tumor formation in the control group (black arrows). H.E. staining. Bar = 1 mm. **B**, The MRI detection revealed the growth of intracranial tumors in the 10 mg/kg SR48692 treatment group and the control group. Scale bar = 5 mm. The T_v_ curve was also showed. ^#^ *p* < 0.01 vs. the control group. Kaplan-Meier survival curve of the glioma-bearing mice in the control group and the SR48692 treatment groups. *p* values vs. the control group are indicated. The MRS analyses in voxels placed in tumor core (blue square) and peritumoral regions (red square) showed the Cho/NAA ratios and Cho/Cr ratios between the SR48692 treatment group and the control group. The *P* values vs. the control group are indicated. **C**, H.E. staining showed the margin of gliomas (black dotted line) in the control group and the 5 mg/kg SR48692 treatment group Scale bar = 100 μm. **D**, NTS immunoreactivity was observed in syngeneic xenografts using IHC staining. The images with a larger magnification in the corner of figures. Scale bar = 100 μm.

To examine the role of NTS and NTSR1 in the progression of glioma *in vivo*, we transplanted GL261 glioma cells into the brains of C57/BL6 mice to establish a syngeneic and orthotopic graft model. Mice received i.p. injection of SR48692 or DMSO every 2 days starting 5 days after tumor cell inoculation, which continued for 3 weeks. 10 days after tumor cell injection, brain swelling appeared at the inoculation sites in the control mice group. However, there was no abnormality in the inoculation sites of the mice treated with 10 mg/kg SR48692 (Figure 6A). Similar results were found in brain tissue following H.E staining, no tumor could be found in the brains of mice treated with 10 mg/kg SR48692 after 10 days of implantation. However, gliomas formed in the brains of the control mice, where glioma cells invaded through the callosum and migrated into the contralateral brain tissue of the inoculation site during the early stages of tumor formation (Figure 6A).

We also assessed MRI detection to monitor the growth of orthotopic xenografts. Tumor dimensions were determined from the MR image and T_v_ was calculated. We found that SR48692 could significantly inhibit the tumor growth *in vivo* (Figure 6B). Kaplan-Meier survival analysis confirmed that treatment with SR48692 significantly prolonged the survival time of the C57/BL6 mice bearing syngeneic GL261 gliomas (median survival of 18.6 ± 3.6 days for DMSO versus 33.0 ± 5.4 days for 10 mg/kg SR48692 treatment) (Figure 6B). Proton magnetic resonance spectroscopy (MRS) can analyze the chemical component in specific tissue region noninvasively. The areas of choline (Cho) peak, n-acetylaspartic acid (NAA) peak and creatine (Cr) peak represent the concentrations of these substances, which reflect the metabolic status of cell and organization. The higher Cho/NAA ratio and Cho/Cr ratio, the more proliferative and malignant in the detected region. In order to evaluate the invasion of xenografts, we carried out MRS detection at the borderline of tumors and found that SR48692 treatment caused a drop in the MRS-detectable Cho/NAA ratio and Cho/Cr ratio in peritumoral tissues (Additional file 7: Figure S5). This meant that there were less malignant cells invading into the preitumoral tissue of SR48692 treatment group (Figure 6B).

Meanwhile, the pathological observation as also performed. In the control group, the margins between the tumor and the normal brain tissue were rough and unclear, indicating an intraparenchymal invasion pattern of glioma. In contrast, the margins of the tumors in the 5 mg/kg SR48692 treatment group were smooth and did not show invasive characteristics of malignant glioma, which were in accord with the MRS detection (Figure 6C). Additionally, we tested the expression of NTS in the xenografts using IHC. NTS was highly expressed in the xenografts of the control group, whereas it was rarely expressed in glioma following SR48692 treatment (Figure 6D). These results indicated that the inhibition of NTSR1 could impair glioma progression *in vivo*.

## Discussion

NTS can be detected in the central nervous system and in the periphery. It produces a wide range of physiological and pharmacological effects [11]. NTS regulates the release of luteinizing hormone and prolactin and has significant interaction with the dopaminergic system, which induces a variety of effects, including: analgesia, hypothermia and increased locomotor activity. In the periphery, neurotensin is found in endocrine cells of the small intestine, where it leads to secretion and smooth muscle contraction. Meanwhile, it also act as a paracrine and endocrine modulator of the cardiovascular system [12,13]. So far, three types of NTS receptors have been discovered, including two G protein coupled receptors (NTSR1 and NTSR2) and a non-specific sorting receptor (NTSR3/sortilin) [13,14]. Recently, it has been shown that NTS is very important in the oncogenic progression of several types of cancer cells [6,7]. NTS has been shown to have growth stimulatory and pro-invasive effects in breast cancer [15,16] and malignant tumors in the digestive system, including hepatomas [17], colon cancer [18,19] and pancreatic cancer [20,21]. Most of its functions in malignant progression are mediated by NTSR1, its high affinity receptor [6]. However, the role of NTS and NTSR1 in malignant glioma has only rarely been reported. Our results confirmed the pro-growth and pro-invasion roles of NTS in glioma progression. NTS and NTSR1 are highly expressed in glioma tissue, especially in glioblastomas, and their expression levels are positively correlated with the pathological grade of the gliomas. We first reported that high levels of NTS and NTSR1 expression predict a decreased survival rate in glioma patients. The proliferation and invasiveness of malignant glioma cells could be suppressed by inhibiting the interaction between NTS and NTSR1 *in vitro*. A similar phenomenon was also observed in the glioma-bearing mice model. SR48692, a NTSR1 specific antagonist, inhibited the invasiveness of orthotopically implanted glioma cells in the mouse brain and prolonged the survival time of the experimental animals. We reported that NTS was expressed at higher levels in glioma stem cells (GSCs) than in differentiated glioma cells [8]. It has been confirmed that GSCs exhibit self-renewal capacity and a high invasive potential, which results in the recurrence of glioma [22]. Considering that NTS can induce neoplastic progression, the role of NTS and NTSR1 in the malignant biological behaviors of GSCs should be investigated in future studies.

In recent decades, it has been shown that inflammation plays an important role in tumorigenesis and tumor progression [23,24]. Intensive infiltration of immune cells and high levels of inflammatory mediators are often found in tumor sites and contribute to malignant biological behaviors. Tumor-associated-microglia/macrophages (TAM/Ms) are the largest population of infiltrating inflammatory cells in glioma [25]. We have reported that NTS played a predominate role in TAM/M recruitment in glioma [8]. It has been confirmed that microglia do not express NTSR1 and NTSR2 but NTSR3, which results in the migration of microglia in a PI3K/MAPK-dependent mechanism [26]. Once recruited to the glioma microenvironment, TAM/Ms promote the proliferation of glioma cells in the stromal areas, enhance the invasiveness of glioma cells at the tumor margins and stimulate angiogenesis in the perivascular areas [27]. Additionally, NTS enhances the release of pro-tumoral inflammation mediators, especially interleukin-8, from tumor cells and inflammatory cells. The enhanced release of IL-8 induced by NTS/NTSR1 has been confirmed in pancreatic cancer [28], hepatocellular carcinomas [17,29] and colon cancer [30,31]. IL-8 widely contributes to the angiogenesis and invasion of tumor cells [31]. Thus, the mechanism of NTS-induced glioma progression is complex and may be divided into three parts. First, NTS can promote the proliferation and invasiveness of glioma cells directly through NTSR1. Second, NTS enhances the release of inflammatory mediators to contribute to glioma progression. Third, it promotes the growth, invasion, angiogenesis and immune evasion of glioma by inducing TAM/M infiltration indirectly through NTSR3.

NTSR1, a seven-transmembrane domain G-protein-coupled receptor, has been shown to bind to G subunits that activate phospholipase C (PLC) [6]. Then, PLC can induce the production of inositol triphosphate (IP3) and stimulate Protein Kinase C (PKC). In various types of tumor cells, the two above-mentioned pathways, especially PKC downstream signaling, regulate the effects of NTS in tumor cells [32]. However, the signaling pathway inducing the malignant properties of NTS in glioma is unclear. Previous researches also confirmed that the effect of NTS/NTSR1 stimulation on cell growth mainly mediated by MEK/ERK1/2 phosphorylation pathway in colon cancer [18] and pancreatic cancer [33]. We found that the MEK1/2-selective inhibitor U0126 could inhibit the proliferation and invasiveness induced by NTS. NTS stimulated Erk1/2 phosphorylation, and the elevated phosphorylation level was reversed by NTS-NA and SR48692. These results indicated that the oncogenic effects of NTS on glioma involved the activation of the MAPK signal pathway. In NTS-stimulated pancreatic cancer cells, two pathways could induce MAPK cascade activation in a PKC-dependent manner [34,35]. PKC could directly stimulate Raf-1, which upregulates MEK/ERK phosphorylation [34]. Meanwhile, PKC could also induce protein kinase D1 (PKD1) activity, which can activate the phosphorylation of Erk1/2 and NF-ΚB [35]. The signaling effector that mediates the activation of the MAPK cascade in NTS-stimulated glioma cells should be investigated further. Additionally, epidermal growth factor receptor (EGFR) transactivation has been reported in NTS-stimulated prostatic cancer cell [36]. Dupouy et al. reported the progression of breast cancer induced by NTS/NTSR1 in an experimental mice model ensues following EGFR, HER2, and HER3 over-expression and autocrine activation [37]. Younes et al. also reported NTS autocrine and/or paracrine regulation causes EGFR, HER2, and HER3 over-expression and activation in lung tumor cells [38]. Prolonged ERK phosphorylation has also been detected in pancreatic cancer cells due to the synergistic stimulation of NTS and EGF [33]. There are at least two pathways through which NTSR1 can mediate the activation of EGFR downstream signaling. First, NTS can induce the release of EGF-like ligands to stimulate EGFR. Meanwhile, NTS can stimulate the phosphorylation of EGFR at Tyr845 by c-Src through a PKC-dependent pathway [39]. High EGFR expression and mutations in EGFR are prevalent in malignant glioma. The amplification and mutation of EGFR has been detected in 40%-50% of GBMs and oligodendrogliomas [40], EGFRvIII, which is a constitutively active EGFR mutant, can be detected in 12%-16% of GBM by IHC. The activation of the EGFR signaling pathway is involved in most of the malignant biological behaviors of glioma. The amplification of EGFR and the expression of EGFRvIII are biomarkers of poor prognosis in glioma patients [41]. It would be very valuable to know whether the cooperative relationship between NTSR1 and EGFR system exists in malignant glioma, and what its underlying molecular mechanism is. Because the NTS/NTSR1-induced transactivation of the EGFR signaling pathway may complicate EGFR-targeted therapies in malignant glioma.

## Materials and methods

### Case selection

Our study was approved by the Ethics Committee of Daping Hospital, Third Military Medical University, Chongqing, P.R. China. Thirty consecutive, surgically resected astrocytomas were identified from the surgical sample database of the Neurosurgery Department of Daping Hospital (Additional file 1: Table S1). None of the patients had undergone chemotherapy or radiotherapy prior to surgery, except two cases of recurrent glioblastoma. All tumor specimens were selected and classified based on the WHO Grade criteria. The peritumoral tissue and relatively normal brain tissue of GBM patient were acquired in fistulization procedure of tumorectomy.

### Analyses of patient data

Gene expression datasets were obtained by R2 microarray analysis and the visualization platform (http://hgserver1.amc.nl/cgi-bin/r2/main.cgi) and Rembrandt database (https://caintegrator.nci.nih.gov/rembrandt/). Affymetrix HU133 Plus 2.0 microarrays were used, the Affymetrix probe-sets for NTS and NTSR1 were 206291_at and 207360_s_at respectively. Kaplan–Meier analysis was conducted online, and the resulting survival curves and P values (log-rank test) were downloaded from internet. All cutoff values for separating the high and low expression groups were determined using the online R2 microarray platform algorithm [9,10].

### Cell culture, drug treatment and siRNA-transfection

The murine glioma cell line GL261 and the human glioma cell line U87 were obtained from the American Type Culture Collection (ATCC, USA) and cultured in DMEM/F12 (Hyclone) supplemented with 10% fetal bovine serum (FBS, Sigma), penicillin and streptomycin (Sigma). The cells were plated and incubated at 37°C to achieve 25–50% confluency. Silencer® Select Pre-Designed siRNA against NTSR1 (NTSR1-siRNA, siRNA ID: 156980 and 143658) and a control-siRNA (negative control #1 siRNA, catalog #: 4390843) were purchased from Ambion (Austin, TX, USA). GL261 cells and U87 cells were transfected with NTSR1-siRNA using Lipofectamine™ RNAiMAX according to the manufacturer’s protocol. After 24 hours, the glioma cells were prepared for related experiments. DMEM/F12 with L-glutamine was used for all serum starvation experiments. The cells were rinsed with phosphate-buffered saline (PBS) and replaced with serum-free medium for 24 hours. For proliferation and 5-bromo-2′-deoxyuridine (BrdU) incorporation experiments, exogenous NTS and/or inhibitors, including the NTS neutralizing antibody (NA-NTS) (2 ng/ml, N2177-01, Biomol, Germany) and SR48692, were added to the medium at the beginning of the serum starvation period. For western blot analysis, the cells were treated with exogenous NTS and/or inhibitors immediately before cell lysis.

### Cell proliferation and DNA synthesis assays

GL261 cells (2×10^3^ cells/well) and U87 cells (2×10^3^ cells/well) were seeded in 96-well plates and serum-starved for 24 hours. Cell proliferation was evaluated using a CCK8 (Cell Counting kit-8) kit according to the manufacturer’s protocol. Briefly, 10 μl CCK8 solution (Dojindo, Kumamoto, Japan) was added to each well, and the samples were incubated at 37°C for 2 hours before the absorbance was measured at 450 nm wave length. Each experimental condition, including blank wells, control wells, and control wells treated with drugs, were assayed in duplicate, and all experiments were performed at least three times.

For DNA synthesis assays, the cells were serum-starved for 24 hours. BrdU assays were performed using BrdU kits (Sigma, St. Louis, MO) as indicated by the manufacturer. Briefly, after serum starvation, BrdU was dissolved in PBS at a final concentration of 1 mg/ml, and 5 μl was added into each well. For each time point, BrdU was mixed into the cells for at least 1 hours, and the cells were stained with primary antibody to BrdU and Cy3-conjugated secondary antibody. The cells were then counterstained with 4′ 6-diamidino-2-phenylindole (DAPI). Fluorescent images were captured using a fluorescence microscope (Carl Zeiss Axio Observer).

### Wound healing and Transwell assays

For the wound healing assays, GL261 cells were plated in 6-well dishes. 24 hours after cells reached 100% confluence, 10 ug/ml mitomycin C was added for 2 hours to eliminate the effect of proliferation, and a scratch was made in the monolayer with a pipette tip. The cells were maintained in low-serum medium (0.1% FCS), and pictures were taken 0, 36 and 72 hours respectively.

Cell invasiveness was studied using a 24-well matrigel transwell chamber assay plate, with an 8 μm pore size membrane (BD Falcon, USA). Matrigel was prepared according to the manufacturer’s instructions. In brief, 10 ug/ml mitomycin C was added to pretreat GL261 cells (5 × 10^4^ cells/100 μl) or U87 cells (5 × 10^4^ cells/100 μl) in serum-free DMEM/F12 medium (Hyclone) for 2 hours. Then, cells were seeded into the upper well of the insert, the lower well was filled with 600 μl of the different conditioned media. After the chambers were incubated at 37°C in a 5%CO_2_ incubator for 20 hours. Invasiveness was calculated by the number of cells invaded through the matrigel chamber and adhered to the bottom of the filter which were stained with crystal violet. Nine fields at 100× magnification were counted for each well. Each experiment was performed in triplicate.

### Elisa, Immunoblotting, immunofluorescence and immunohistochemistry

The NTS peptide levels in glioma were measured by the ELISA method (CUSABIO’ Human Neurotensin ELISA Kit), according to the manufacturer’s instructions. 100 mg tissue was rinsed with 1X PBS, homogenized in 1 ml of 1X PBS and stored overnight at −20°C. After two freeze-thaw cycles were performed to break the cell membranes, the homogenates were centrifuged for 5 minutes at 5000 × g, 2 - 8°C. The supernate was removed and assayed immediately. The limit of detection was 15.6 pg/ml-1000 pg/ml with specificity that recognizes both natural and recombinant human NTS and sensitivity of < 3.9 pg/ml. There were three duplicative holes for every sample in Elisa assay.

Immunoblotting was conducted according to the standard procedures outlined in the Additional file 8. The membrane was incubated with antibodies against ProNTS (1:500; N2177-10, Biomol, Germany) or NTSR1 (1:1000; ab117592, Abcam, USA) at 4°C for 12 h. Horseradish peroxidase-conjugated goat anti-mouse and goat anti-rabbit immunoglobulin G (1:500; A0216&A0208, Beyotime, China) were used as secondary antibodies. Proteins were visualized using a Super Signal West Pico chemiluminescence kit (Pierce) and were quantified using the Odyssey system and software (LI-COR Biosciences). For immunofluorescence, the cells were fixed with 4% paraformaldehyde (PFA). Two kinds of primary antibodies for NTSR1 (ab183088 & ab117592, Abcam,USA) were used at 1:300 dilution on GL261 cells and U87 cells. Cy3-labeled goat anti-mouse IgG (1:500; A0521, Beyotime, China) for GL261 and FITC-labeled goat anti-rabbit IgG (1:500; A0562, Beyotime, China) for U87 were used as secondary immunofluorescence antibodies. The nuclei were stained with DAPI. Fluorescent images were captured using a fluorescence microscope (Carl Zeiss Axio Observer).

Immunohistochemistry (IHC) was performed on paraffin-embedded sections. The tumor sections were incubated with primary antibodies for NTS (1:500, N2177-01, Biomol, Germany) and for NTSR1 (1:200, ab117592, Abcam, USA), followed by detection using a ChemMate Detection kit (Dako, Denmark). A positive reaction was indicated by brown color using DAB, and the cells were counterstained with hematoxylin.

### Syngeneic orthotopic glioma implantation and Magnetic resonance imaging (MRI) experiments

All procedures involving mice were conducted in accordance with the Guidelines of Animal Experiments of Third Military Medical University. All mice were purchased from Experimental Animal Center of Third Military Medical University. GL261 cells (5 × 10^4^) were injected orthotopically into the brains of 6-week old female C57BL/6 mice (n = 24). The detailed measurement of intracranial tumors was listed in Additional file 8. 3 days after injection, 20 mice were randomly divided into 4 groups of 5 animals each. The groups were treated through i.p. injection with 2 mg/kg, 5 mg/kg, 10 mg/kg SR48692 respectively. SR48692 was resuspended in DMSO which was used as control. The mice were treated every two days for a total of five times. The weigh curves in different groups were recorded and showed in Additional file 5: Figure S3E. The survival periods of the mice were recorded. The brains of the mice were collected, fixed in formalin, and paraffin-embedded. The rest 4 mice were also divided into two groups at 3 days after implantation and treated with DMSO or SR48692 (10 mg/kg) respectively. 10 days after tumor cell injection, these 4 mice were euthanized and their brains were removed and processed for histopathologic analysis. The MRI equipment was a Bruker Biospec 7.0 Tesla imaging system (Bruker BioSpin MRI GmbH, Germany). MRI experiments were carried out under general anesthesia (1–2% isoflurane, 0.8–1.0 L/min O_2_). Mice were imaged at 7 days after the cells were injected and then every 3 days until 19 days after implantation. Tumor dimensions were determined from the MR image and tumor volume (T_v_) was calculated using the formula: T_v_ = (π/6) × length × width × depth. MRS was used to monitor the glioma invasion. We placed a voxel (1 mm × 1 mm × 1 mm) at the borderline of tumors and repeated the MRS test for several times. Cho/NAA ratios and Cho/Cr ratios in these regions were calculated to assess the invasion of glioma cells in peritumoral tissues.

### Statistical analysis

All statistical analyses were performed using the SPSS 13.0 statistical package (SPSS, Chicago, IL, USA). The statistical significance of the differences among more than three groups was determined using one way analysis of variance (ANOVA) with Bonferroni’s Multiple Comparison to compare each two groups. The statistical significance of the differences between two groups was determined using a t test. Survival data were analyzed using the log-rank test. Differences were considered significant when *p* < 0.05.

### Main points

- Expression levels of NTS/NTSR1 positively correlated with glioma pathological grade.
- High expression levels of NTS/NTSR1 indicate a worse prognosis in glioma patients.
- NTS/NTSR1 signaling regulates the proliferation and invasiveness of glioma cells.
- NTSR1 antagonist, SR48692, prolongs the survival periods of glioma-bearing mice.

## Additional files

Additional file 1: Table S1. Summary of specimens examined. Additional file 2: Table S2. The quantitative analyses of NTS and NTSR1 expression derived from IHC staining. Additional file 3: Figure S1. Western blot analysis of NTS and NTSR1 expression in glioma samples. A, Western blot analysis of NTSR1 expression in 30 glioma specimens. B, Western blot analysis of NTS expression in 30 glioma specimens. Additional file 4: Figure S2. The prognostic value of NTS in TCGA database and Rembrandt database. A, NTS had a significantly negative relationship with the progression-free survival probability of de novo GBM patients. B, The analyses from Rembrandt database confirmed that high expression level of NTS indicated a significantly worse prognosis in several sub-databases, especially in “Astrocytoma” sub-database. Additional file 5: Figure S3. Representative IHC staining controls and NTSR1-siRNA knockdown in glioma cells. A, ProNTS and NTSR1 mRNA can be both detected in GL261 and U87 cells by quantitative Realtime-PCR. The mRNA expression levels in glioma cell lines were significantly lower than their expressions in primary glioma cells (G11 and G13). B, The representative IHC staining controls for NTS and NTSR1 immunoreactivies were shown. C, The cell apoptosis levels of glioma cell lines on dose of 10 μM SR 48692 in FACS assay. D, Western blot analysis confirmed that NTSR1-siRNA effectively knocked down NTSR1 expression in GL261 cells and U87 cells. E, The weight of mice were recorded regularly during the in vivo experiments. The weight of mice increased steadily during the experiment, but dropped rapidly in the several days before death. Additional file 6: Figure S4. Activated status analysis of signaling molecules after NTS stimulation. Raf-1/Mek/Erk1/2 pathway was activated after NTS stimulation, but not other molecules and pathways, including p15, p16, p38, pAKT, mTOR, pSmad2/3, pJAK2 and pSTAT3. Additional file 7: Figure S5. The curves of MRS analysis in tumor tissue and peritumoral tissue of the syngeneic orthotopic glioma. Additional file 8:
**Immunofluorescence and Immunohistochemistry.**
