# Correction: Neurotensin promotes the progression of malignant glioma through NTSR1 and impacts the prognosis of glioma patients

**DOI:** 10.1186/s12943-022-01632-7

**Published:** 2022-08-10

**Authors:** Qing Ouyang, Xueyang Gong, Hualiang Xiao, Ji Zhou, Minhui Xu, Yun Dai, Lunshan Xu, Hua Feng, Hongjuan Cui, Liang Yi

**Affiliations:** 1 Department of Neurosurgery, Daping Hospital, Third Military Medical University, Chongqing, China; 2 State Key Laboratory of Silkworm Genome Biology, Institute of Sericulture and Systems Biology, Southwest University, Chongqing, China; 3 Department of Pathology, Daping Hospital, Third Military Medical University, Chongqing, China; 4 Division of Hematology/Oncology, Department of Medicine, Virginia Commonwealth University, Richmond, VA USA; 5 Department of Neurosurgery, Southwest Hospital, Third Military Medical University, Chongqing, China

**Correction: Mol Cancer 14, 21 (2015)**

**https://doi.org/10.1186/s12943-015-0290-8**

Following publication of the original article [1], the authors subsequently identified an error in Figure 4A. The images representing U87 cells were taken from GL261 cells by mistake. The corrected Figure 4 is now shown in this correction. The authors confirm that the conclusions of this article are not affected, and sincerely apologize for this error and any inconvenience that may have caused. The correct figure is as follows.

**Fig. 4:**
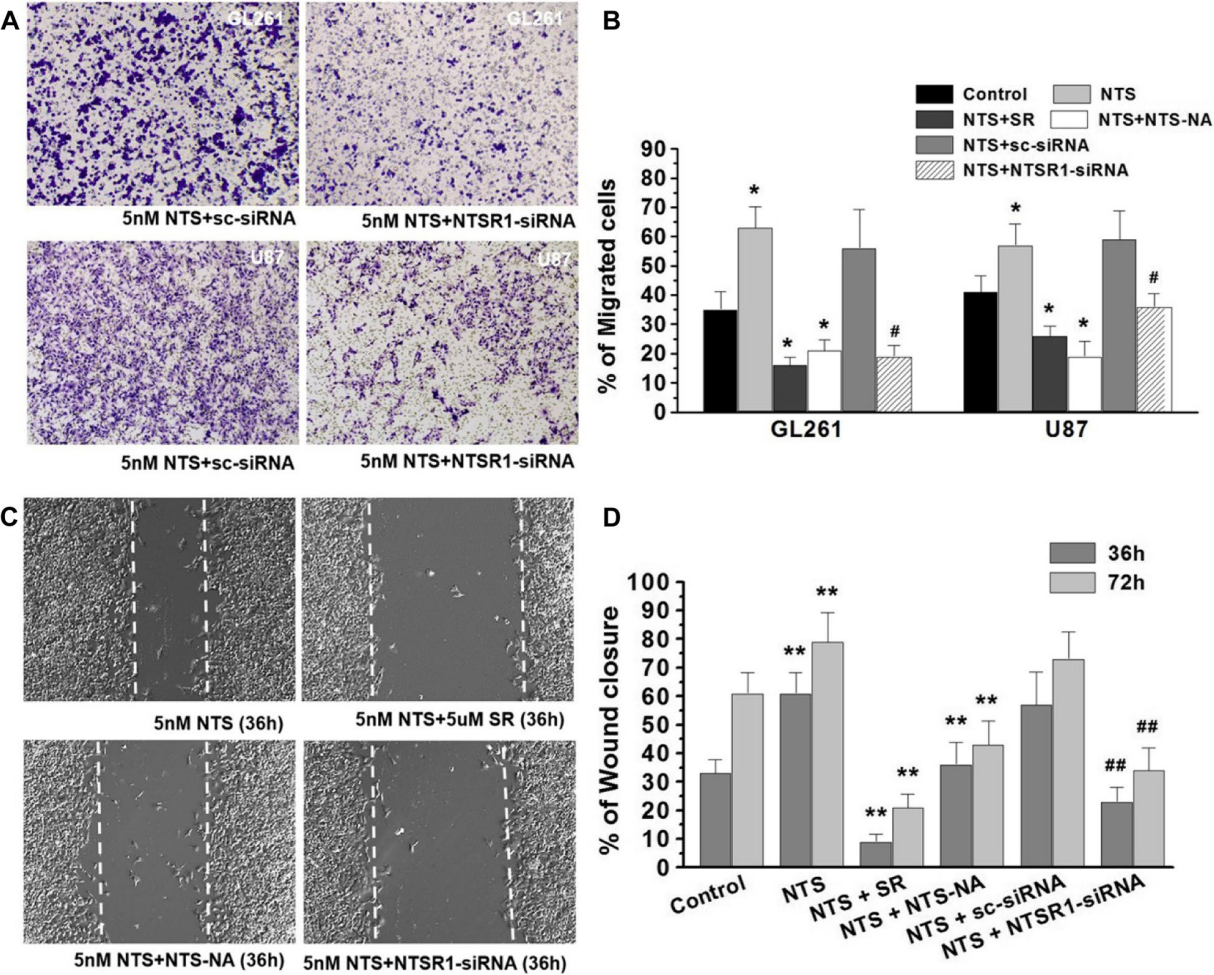
NTS/NTSR1 boosted the migration capacity and invasiveness of glioma cells. **A**, Transwell invasion assay showed that the ability of GL261 and U87 glioma cells to invade across the matrigel and membrane. **B**, The proportion of invasive GL261 and U87 glioma cells in all the experimental groups in the transwell experiments. *p < 0.01 vs. the control group using a two-tailed t test, & *p* < 0.01 vs. the NTS group using a two-tailed t test. ^#^ *p* < 0.01 vs. the NTS + sc-siRNA group using a two-tailed t test. **C**, Illustrations of the scratch wounds inflicted by a pipette tip. After 36 hours, the scratch wounds were recolonized by GL261 cells cultured in low-serum medium (0.1% FCS). **D**, The percentage of wound closure by the cells was quantified in the different groups at 36 hours and 72 hours. && *p* < 0.01 vs. the NTS group using a two-tailed t test. **p < 0.01 vs. the control group using a two-tailed t test, ^##^ *p* < 0.01 vs. the NTS + sc-siRNA group using a two-tailed t test

## References

[1] Ouyang Q, Gong X, Xiao H (2015). Neurotensin promotes the progression of malignant glioma through NTSR1 and impacts the prognosis of glioma patients. Mol Cancer.

