# Supplementary material for: Neurotensin promotes the progression of malignant glioma through NTSR1 and impacts the prognosis of glioma patients
Source: Mol Cancer. 2015 Feb 3;14:21. doi: 10.1186/s12943-015-0290-8 (PMC4351837; doi:10.1186/s12943-015-0290-8)
Supplement: Additional file 1: Table S1. — Summary of specimens examined. [file 12943_2015_290_MOESM1_ESM.docx]

| **Table S1. Summary of specimens examined** | | | | | | |
| --- | --- | --- | --- | --- | --- | --- |
| Patient no. | Code | Age(yr)/Sex | Histology/WHO Grade | Location | NTS positive glioma cells (%) | NTSR1 positive glioma cells (%) |
| 1 | G20 | 33/F | DA/II | Temporal L | 5.3±1.7 | 18.1±2.9 |
| 2 | G73 | 16/F | DA/II | Parietal L | 22.9±6.1 | 7.6±2.1 |
| 3 | G86 | 68/M | DA/II | Frontal L | 13.7±4.4 | 6.1±3.2 |
| 4 | G36 | 27/F | DA/II | Frontal L | 6.1±2.5 | 3.3±1.6 |
| 5 | G47 | 44/F | DA/II | Parietotemporal R | 21.6±6.1 | 15.7±4.3 |
| 6 | G37 | 26/F | DA/II | Temporal L | 3.6±1.1 | 22.3±7.1 |
| 7 | G39 | 49/F | DA/II | Frontoparietal R | 2.3±1.1 | 6.1±0.9 |
| 8 | G21 | 52/M | DA/II | Frontal L | 6.2±1.6 | 16.4±3.5 |
| 9 | G98 | 25/M | DA/II | Frontotemporal L | 18.3±4.1 | 13.2±5.6 |
| 10 | G43 | 30/M | DA/II | Temporal L | 32.5±8.6 | 9.2±3.7 |
| 11 | G71 | 49/F | AA/III | Temporal R | 36.2±8.6 | 36.8±8.3 |
| 12 | G62 | 54/F | AA/III | Temporal L | 23.8±6.5 | 70.8±12.3 |
| 13 | G33 | 67/M | AA/III | Temporal L | 8.8±3.5 | 17.5±5.9 |
| 14 | G06 | 44/M | AA/III | Occipital R | 10.6±3.3 | 21.2±3.9 |
| 15 | G16 | 40/M | AA/III | Temporal L | 33.7±7.1 | 55.3±10.2 |
| 16 | G80 | 31/M | AA/III | Frontal R | 7.8±2.6 | 19.0±4.1 |
| 17 | G29 | 61/M | AA/III | Frontoparietal R | 33.1±3.9 | 43.3±12.5 |
| 18 | G19 | 62/M | AA/III | Temporal L | 9.7±2.5 | 15.1±2.3 |
| 19 | G11 | 63/M | AA/III | Parietotemporal R | 7.3±3.9 | 63.9±8.5 |
| 20 | G07 | 32/F | AA/III | Frontal L | 49.2±11.6 | 51.6±8.7 |
| 21 | G13 | 53/F | pGBM/IV | Frontal L | 89.1±3.5 | 91.8±3.3 |
| 22 | G30 | 40/M | pGBM/IV | Temporal L | 23.6±5.3 | 46.7±6.5 |
| 23 | G63 | 48/F | pGBM/IV | Parietal R | 51.3±5.9 | 33.2±5.9 |
| 24 | G14 | 52/F | pGBM/IV | Temporal L | 43.5±11.3 | 65.8±13.5 |
| 25 | G10 | 57/M | pGBM/IV | Parietooccipital R | 91.1±3.7 | 96.2±1.7 |
| 26 | G95 | 50/F | pGBM/IV | Frontoparietal R | 82.6±13.9 | 89.3±6.5 |
| 27 | G61 | 40/F | pGBM/IV | Temporal L | 96.6±2.5 | 93.7±2.3 |
| 28 | G74 | 44/M | pGBM/IV | Occipital R | 75.5±6.7 | 81.1±16.2 |
| 29 | G32 | 40/F | sGBM/IV | Frontal L | 32.4±7.1 | 12.7±3.6 |
| 30 | G53 | 38/M | sGBM/IV | Frontotemporal L | 78.1±9.2 | 52.1±16.6 |
| Abbreviations: M, male; F, female; WHO, World Health Organization; DA, diffuse astrocytoma; AA, anaplastic astrocytoma; pGBM, primary glioblastoma; sGBM, secondary glioblastoma; R, right; L, Left | | | | | | |
