# Supplementary material for: Neurotensin promotes the progression of malignant glioma through NTSR1 and impacts the prognosis of glioma patients
Source: Mol Cancer. 2015 Feb 3;14:21. doi: 10.1186/s12943-015-0290-8 (PMC4351837; doi:10.1186/s12943-015-0290-8)
Supplement: Additional file 2: Table S2. — The quantitative analyses of NTS and NTSR1 expression derived from IHC staining. [file 12943_2015_290_MOESM2_ESM.docx]

| **Table S2. The quantitative analyses of NTS and NTSR1 expression derived from IHC staining** | | | | | | | | | |
| --- | --- | --- | --- | --- | --- | --- | --- | --- | --- |
| Histology/WHO Grade | Percentage of NTS positive glima cells | | | | Percentage of NTSR1 positive glima cells | | | | |
|  | n | % | 95%CI | *P* Value | n | % | 95%CI | | *P* Value |
| GBM/IV | 10 | 66.4±26.4 | 47.5-85.2 | *p* = 0.001 vs AA  *p* < 0.001 vs DA | 10 | 66.3±29.1 | 45.5-87.0 | | *p* = 0.087 vs AA  *p* = 0.001 vs DA |
| AA/III | 10 | 22.0±15.2 | 11.2-32.9 | *p* = 0.001 vs GBM  *p* = 0.385 vs DA | 10 | 39.5±20.6 | 24.7-54.2 | | *p* = 0.087 vs GBM  *p* = 0.006 vs DA |
| DA/II | 10 | 13.3±10.2 | 6.0-20.5 | *p* < 0.001 vs GBM  *p* = 0.385 vs AA | 10 | 11.8±6.24 | 7.3-16.3 | | *p* = 0.001 vs GBM  *p* = 0.006 vs AA |
| Total | 30 | 33.9±29.7 |  | | 30 | 39.2±30.3 | |  | |
