# Supplementary figures and images for: Neurotensin promotes the progression of malignant glioma through NTSR1 and impacts the prognosis of glioma patients
Source: Mol Cancer. 2015 Feb 3;14:21. doi: 10.1186/s12943-015-0290-8 (PMC4351837; doi:10.1186/s12943-015-0290-8)

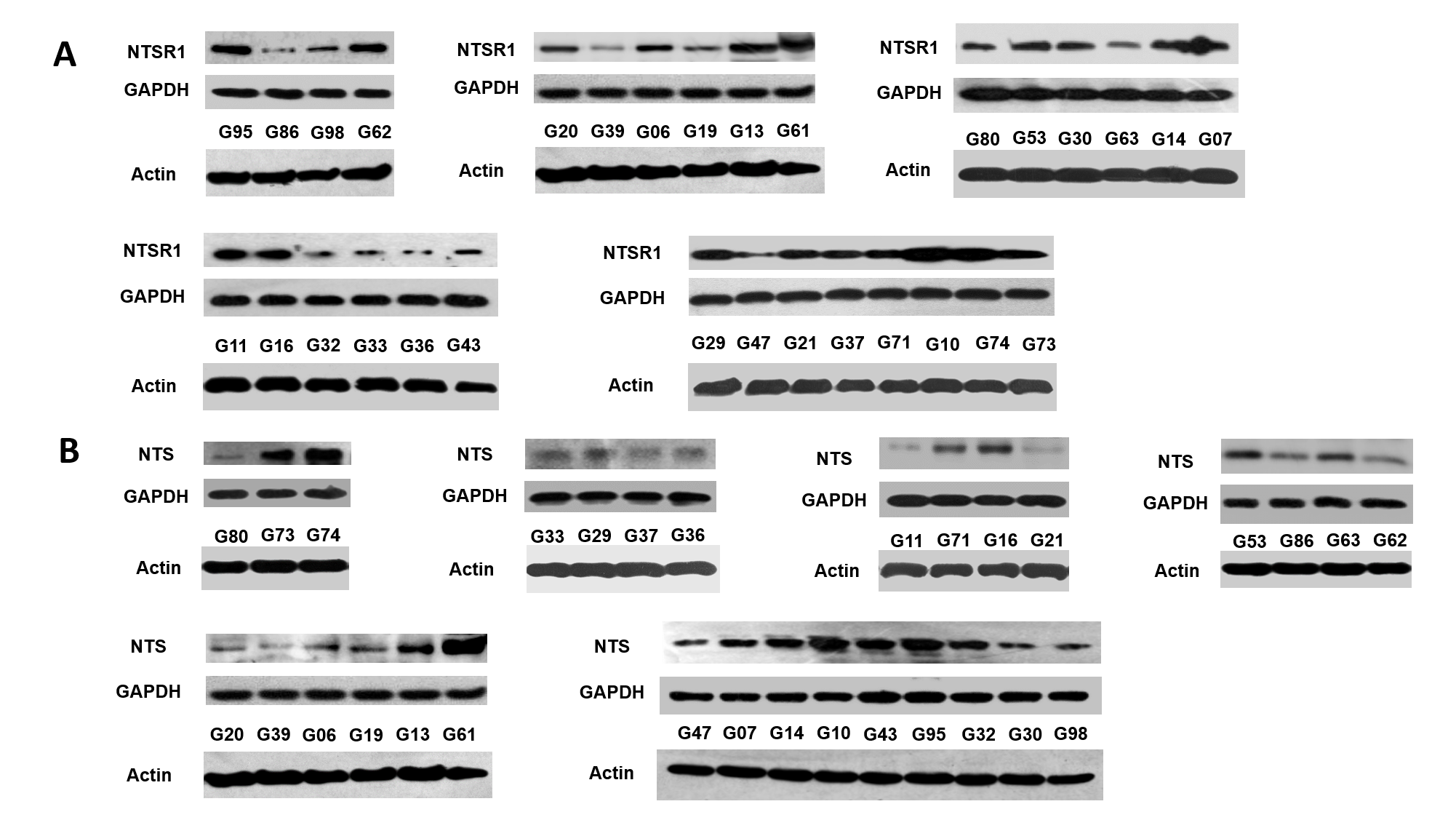

Supplement: Additional file 3: Figure S1. — Western blot analysis of NTS and NTSR1 expression in glioma samples. A, Western blot analysis of NTSR1 expression in 30 glioma specimens. B, Western blot analysis of NTS expression in 30 glioma specimens. [file 12943_2015_290_MOESM3_ESM.tiff]

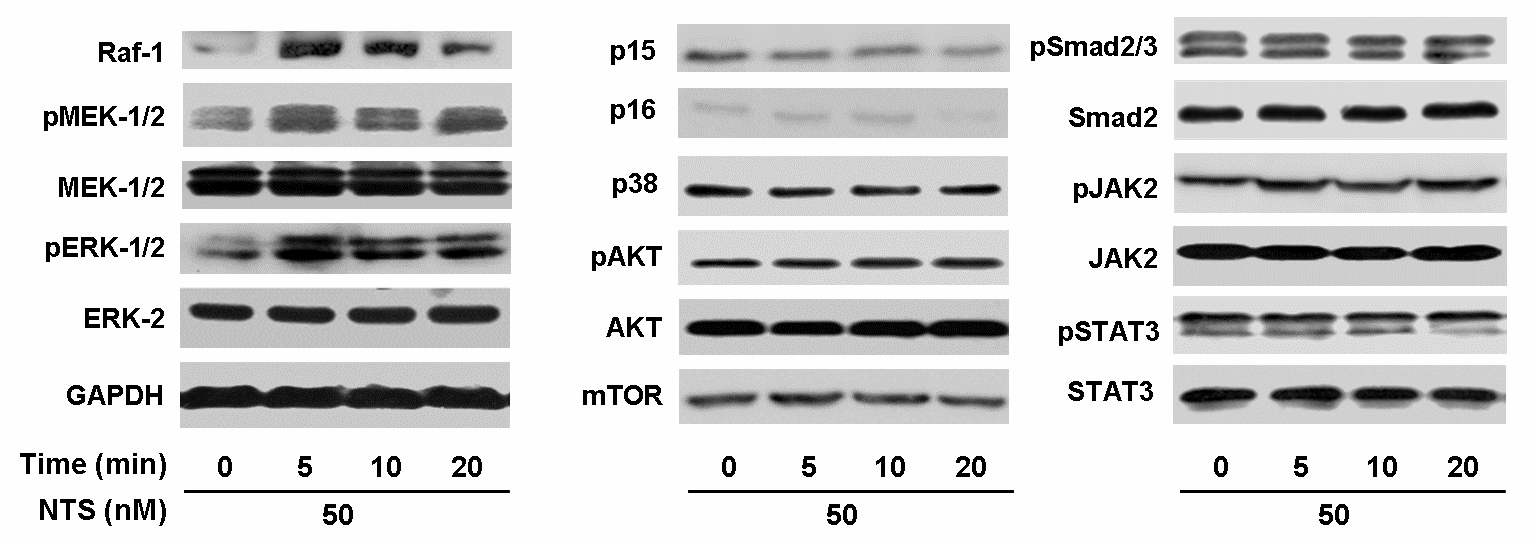

Supplement: Additional file 4: Figure S2. — The prognostic value of NTS in TCGA database and Rembrandt database. A, NTS had a significantly negative relationship with the progression-free survival probability of de novo GBM patients. B, The analyses from Rembrandt database confirmed that high expression level of NTS indicated a significantly worse prognosis in several sub-databases, especially in “Astrocytoma” sub-database. [file 12943_2015_290_MOESM4_ESM.tiff]

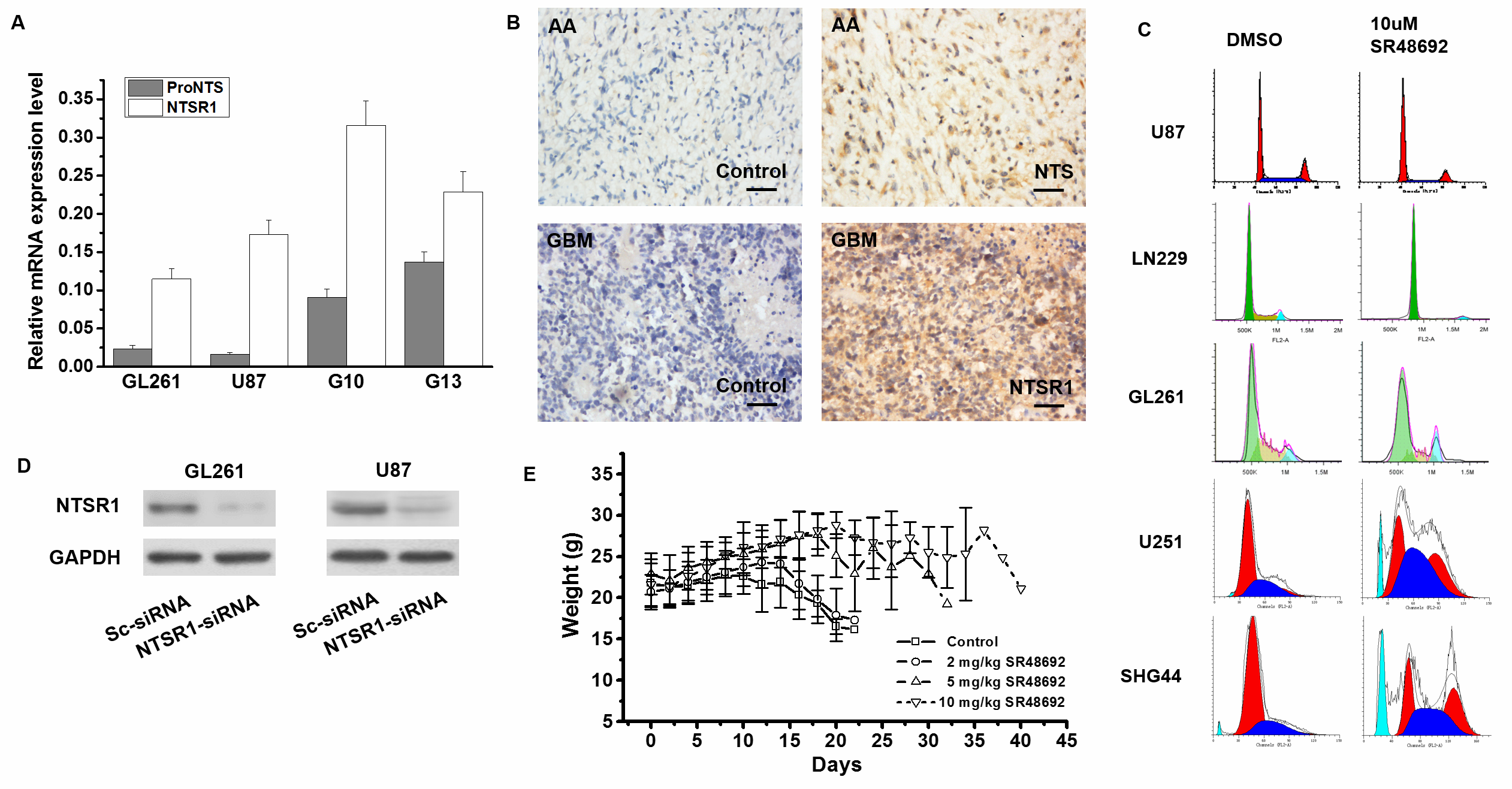

Supplement: Additional file 5: Figure S3. — Representative IHC staining controls and NTSR1-siRNA knockdown in glioma cells. A, ProNTS and NTSR1 mRNA can be both detected in GL261 and U87 cells by quantitative Realtime-PCR. The mRNA expression levels in glioma cell lines were significantly lower than their expressions in primary glioma cells (G11 and G13). B, The representative IHC staining controls for NTS and NTSR1 immunoreactivies were shown. C, The cell apoptosis levels of glioma cell lines on dose of 10 μM SR 48692 in FACS assay. D, Western blot analysis confirmed that NTSR1-siRNA effectively knocked down NTSR1 expression in GL261 cells and U87 cells. E, The weight of mice were recorded regularly during the in vivo experiments. The weight of mice increased steadily during the experiment, but dropped rapidly in the several days before death. [file 12943_2015_290_MOESM5_ESM.tiff]

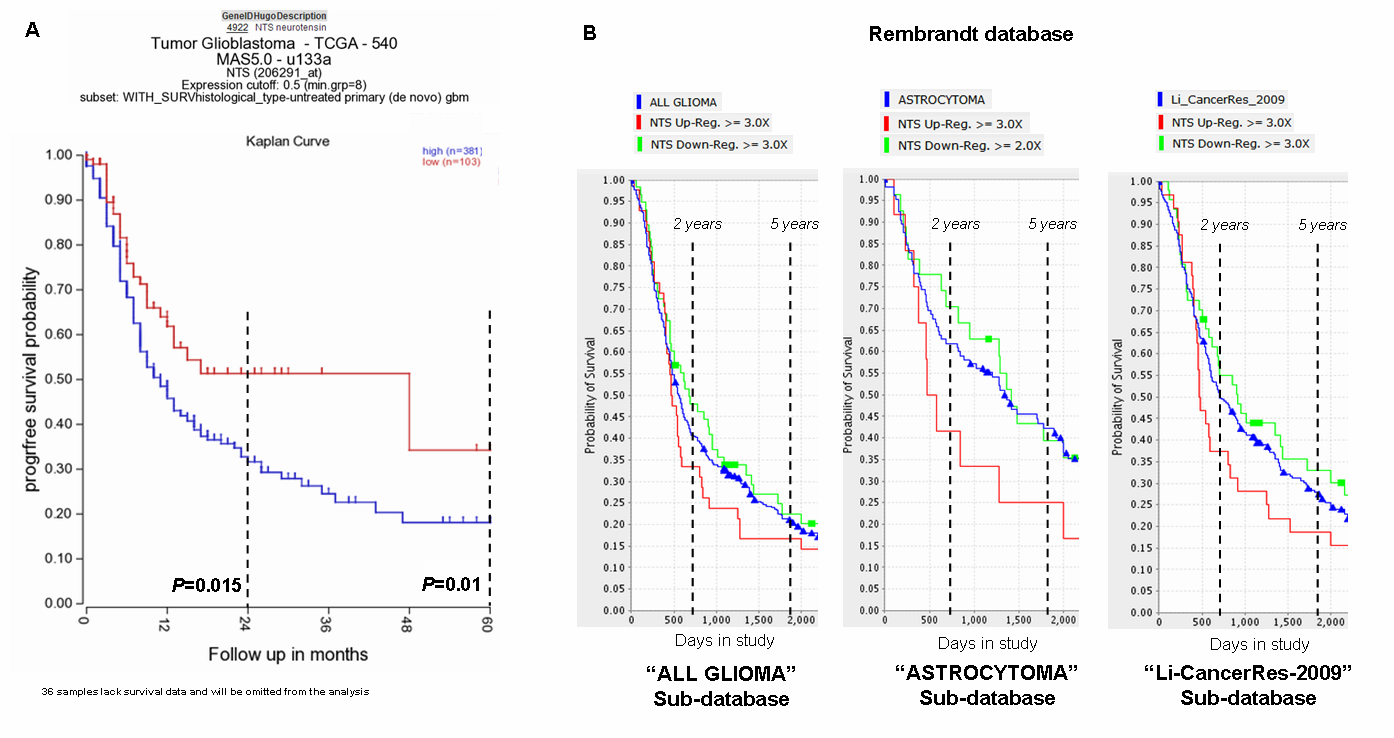

Supplement: Additional file 6: Figure S4. — Activated status analysis of signaling molecules after NTS stimulation. Raf-1/Mek/Erk1/2 pathway was activated after NTS stimulation, but not other molecules and pathways, including p15, p16, p38, pAKT, mTOR, pSmad2/3, pJAK2 and pSTAT3. [file 12943_2015_290_MOESM6_ESM.tiff]

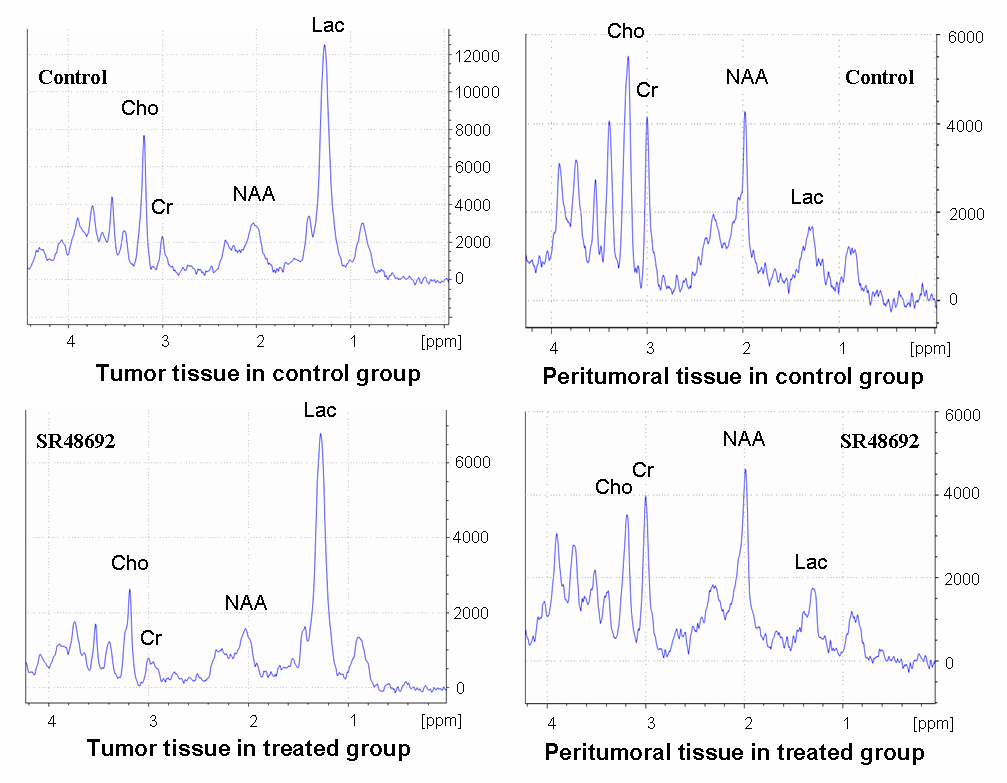

Supplement: Additional file 7: Figure S5. — The curves of MRS analysis in tumor tissue and peritumoral tissue of the syngeneic orthotopic glioma. [file 12943_2015_290_MOESM7_ESM.tiff]
