# Supplementary material for: Neurotensin promotes the progression of malignant glioma through NTSR1 and impacts the prognosis of glioma patients
Source: Mol Cancer. 2015 Feb 3;14:21. doi: 10.1186/s12943-015-0290-8 (PMC4351837; doi:10.1186/s12943-015-0290-8)
Supplement: Additional file 8: — Immunofluorescence and Immunohistochemistry. [file 12943_2015_290_MOESM8_ESM.doc]

**Supplemental materials and methods:**

**Immunofluorescence and Immunohistochemistry**

Cell immunofluorescent staining of NTSR1 was performed by using standard techniques. Cells were grown on collagen-coated coverslips in 24 well cell culture plate through the night. After the cells were attached in the coverslips, wash the cells with phosphate-buffered saline (PBS), fixed for 15 min in 4% paraformaldehyde in PBS. The cells were blocked with 10% milk in PBS for 1 h, incubated with the primary antibody in blocking buffer for 2 h at 37 ℃, washed in PBS, and then incubated with the appropriate secondary antibody for 2h at room temperature. Two kind of primary antibodies for NTSR1 (ab183088 & ab117592, Abcam, USA) were used at 1:300 dilution on GL261 cells and U87 cells. Cy3-labeled goat anti-mouse IgG (1:500; A0521, Beyotime, China) for GL261 and FITC-labeled goat anti-rabbit IgG (1:500; A0562, Beyotime, China) for U87 were used as secondary immunofluorescence antibodies. Subsequently, Cells were counterstained with DAPI (Beyotime, 1:100) to stain nuclei and incubated for 15 min at room temperature. Fluorescent images were captured with a fluorescence microscope (Carl Zeiss Axio Observer). For immunohistochemistry, paraffin embedded tumor tissues were sectioned at 5μm, deparaffinized, and rehydrated. For antigen retrieval, sections were treated for 20 minutes at 95 °C in 10 mmol/L citrate buffer (pH 6.0) in a laboratory microwave oven, and subsequently washed, blocked and incubated at 4 ℃ overnight with primary antibodies for NTS (1:500; N2177-01, Biomol, Germany,) or NTSR1 (1:200, ab117592, Abcam, USA). On the next day, slides were incubated with HRP-labeled secondary antibody (Dako, Denmark) for 2 hours at room temperature. The antigen-antibody complex was revealed with 3,3'-diaminobenzidine tetrahydrochloride (Dako, Denmark) complex for 5 minutes according to the manufacturer’s instructions, and counterstained with hematoxylin. Sections were visualized using a Nikon Upright Metallurgical microscope.

**Western-blot**

Protein extracts were fractionated by SDS-PAGE and transferred to a polyvinylidene difluoride membrane using a transfer apparatus. After incubation with 5% nonfat milk in TBST (0.1 mM Tris-HCl, pH 8.5, 150 mM NaCl, 0.5% Tween 20) for 60 min, the membrane was washed once with TBST and incubated with antibodies against ProNTS (1:500; N2177-10, Biomol, Germany) or NTSR1 (1:1000; ab117592, Abcam, USA) at 4 °C for 12 h. Membranes were washed three times for 10 min and incubated with a 1:500 dilution of horseradish peroxidase-conjugated secondary antibodies (A0216&A0208, Beyotime, China) for 2 h with 37 ℃. Blots were washed with TBST three times and signals were visualized using enhanced chemiscope detection reagent from Clinx Science Instruments, and captured by the ECL Western blotting detection system (Bedford, MA, USA).

**Intracranial tumors establishment**

Glioma cells were trypsinized, washed twice in phosphate-buffered saline, then resuspended in phosphate-buffered saline at 5 × 10^3^ cells/μl and kept on ice until injected intracranially into mice. For this procedure, the calvarium of each anesthetized mouse was exposed through a midline incision, and a burr hole was drilled 1 mm lateral (right) and 2 mm anterior to the bregma. Utilizing a small animal stereotactic frame (RWD Life Science, Shenzhen, China.), cells were injected at a rate of 1 μl/min for 10 min (total of 5 × 10^4^ cells injected per mouse), with the syringe left in place for 10 min following completion of tumor cell injection. Injections were to a depth of 3 mm below the outer table of the skull, thereby introducing the tumor cells near the right caudate nucleus.
